# Nanoparticles-based anti-aging treatment of Alzheimer’s disease

**DOI:** 10.1080/10717544.2022.2094501

**Published:** 2022-07-19

**Authors:** Jian-Jian Chu, Wen-Bo Ji, Jian-Hua Zhuang, Bao-Feng Gong, Xiao-Han Chen, Wen-Bin Cheng, Wen-Danqi Liang, Gen-Ru Li, Jie Gao, You Yin

**Affiliations:** a Second Affiliated Hospital (Changzheng Hospital) of Naval Medical University, Shanghai, China; b Changhai Clinical Research Unit, Shanghai Changhai Hospital, Naval Medical University, Shanghai, China

**Keywords:** AD, anti-aging, BBB, nanoparticles, senescent cells, anti-inflammation

## Abstract

Age is the strongest risk factor for Alzheimer’s disease (AD). In recent years, the relationship between aging and AD has been widely studied, with anti-aging therapeutics as the treatment for AD being one of the mainstream research directions. Therapeutics targeting senescent cells have shown improvement in AD symptoms and cerebral pathological changes, suggesting that anti-aging strategies may be a promising alternative for AD treatment. Nanoparticles represent an excellent approach for efficiently crossing the blood-brain barrier (BBB) to achieve better curative function and fewer side effects. Thereby, nanoparticles-based anti-aging treatment may exert potent anti-AD therapeutic efficacy. This review discusses the relationship between aging and AD and the application and prospect of anti-aging strategies and nanoparticle-based therapeutics in treating AD.

## 1. Introduction

AD is the most common form of dementia, accounting for about 60–80% of all dementia cases (Alzheimer’s Association, 2021). AD can lead to impaired cognitive functions such as memory, language, and learning, the decline of executive function, and mental symptoms. Age is one of the strongest risk factors for AD (Hebert et al., 2010). In China, 18.70% of the population is more than 60 years of age (Communiqué of the Seventh National Population Census). More than one-fifth of this population suffers from mild cognitive impairment (MCI) and dementia, and 3.9% is the case rate of AD (Jia et al., 2020). With the aging population, many AD patients and those suffering from dementia and MCI will become a heavy health and economic burden for individuals, families, and societies all over the world in the foreseeable future (Jia et al., 2020; Alzheimer’s Association, 2021). Hence, many studies have focused on the treatment of AD.

Based on the cholinergic hypothesis and excitotoxicity hypothesis, the commonly used drugs for the treatment of AD include acetylcholinesterase inhibitors and noncompetitive N-methyl-D-aspartic acid (NMDA) receptor antagonists, which show moderate improvement in symptoms (Livingston et al., 2020) but do not delay or stall the damage and destruction of neurons (Alzheimer’s Association, 2021). Currently, the disease-modifying treatments (DMTs) for AD are urgently needed. According to the prevailing viewpoints, β-amyloid (Aβ) deposition and the abnormal hyperphosphorylation of tau proteins serve as the main pathogenesis and pathological features of AD (Bloom, 2014; Sengupta et al., 2016). Targeting Aβ protein has always been one of the cores of AD research and treatment development (Nalivaeva & Turner, 2019). Compared with Aβ, cognitive impairment and neuron loss are closely correlated with abnormal hyperphosphorylation of tau protein (Nelson et al., 2012). Strategies for targeting tau protein are always the research hotspots. Nevertheless, there are still no significantly effective DMTs for AD until now.

Due to the distinctiveness of the age of onset of AD, aging has always been closely related to the disease. Aging can be defined as the decrease in the psychological and physiological adaptability of the body to the environment, eventually leading to death. One of the features of aging is the accumulation of senescent cells in many tissues (Campisi, 2011). In addition to the decline of normal functions, the other causes of senescence leading to aging-related diseases are also associated with the secretion of senescence-related secretory phenotype (SASP) from senescent cells, which induce the damage of the adjacent cells and change the microenvironment around them (Childs et al., 2015). Senescent cells of the cerebrum, especially senescent astrocyte and senescent microglia, play an important role in the development and progression of AD and have been studied widely. Although the exact mechanism of senescence cells and AD has not been well expounded, anti-aging strategies have exhibited great potential and can become an effective treatment avenue for AD (Kritsilis et al., 2018).

The BBB hinders the entry of many agents into the CNS, including most anti-aging drugs. There are many strategies to increase the stability and efficiency of anti-senescent drugs and lower their loss while penetrating BBB, and one of the promising choices is nanoparticles. Nanoparticles can be of different types (e.g. lipid-based NPs, polymeric NPs, inorganic NPs, and nanogels) and can assist drugs in entering the CNS by bypassing or directly passing through the BBB. In this review, we mainly discuss nanoparticles that directly penetrate the BBB. They can cross over the BBB by four main pathways, including carrier-mediated transport (CMT), receptor-mediated transcytosis (RMT), adsorptive-mediated transcytosis (AMT), and cell-mediated transport (Furtado et al., 2018; Bilal et al., 2020).

## 2. Aging and AD

The nervous system is a dynamically balanced integration of a wide range of functional cells. Typically, most neurodegenerative diseases are manifested in the elderly (Grimm & Eckert, 2017). Aging is the most important risk factor for the development of neurodegenerative diseases. The incidence rate of AD has increased with increasing age (Jia et al., 2020). The primary mechanism by which aging promotes the process of AD may be attributed to the abnormal functioning of senescent cells and the persistent chronic inflammation in the CNS induced by the active secretion of SASP by senescent cells (Franceschi & Campisi, 2014; Heneka et al., 2015; Birch & Gil, 2020), which finally results in decreased synaptic function or neuron loss. However, the causal relationship between aging and AD is not clear. In this review, we search for the clues of aging in AD from three aspects: aging glial cells, aging neurons and neural stem cells, and aging BBB.

### 2.1. Aging glial cells

Several studies have focused on the senescence of microglia, astrocytes, oligodendroglia, and oligodendrocyte progenitor cells (OPCs) for their crucial roles in neuroprotection, and maintenance of CNS homeostasis (ion concentration, neurotransmitters, inflammatory mediator, etc.), and the brain immune system (Jessen, 2004).

Microglia are the primary immune cells in CNS and are usually in a static state. They are similar to macrophages in function, secrete various growth factors and cytokines, and stimulate phagocytosis in response to invasive pathogens or damage (Nayak et al., 2014). Microglia play critical roles in maintaining CNS homeostasis by clearing misfolded proteins and controlling inflammation (Mhatre et al., 2015). They can accumulate around Aβ deposits and then phagocytize and clear Aβ (Yeh et al., 2016). Human genetic studies have identified microglia as a key cell type to control the risk of AD. Genetic risk variants of AD such as APOE4, TREM2, CD33, ABCA7, HLA-DRB1/B5, and INPP5D were identified as moderately or highly expressing in microglia via human genome-wide association studies (GWAS) (Mhatre et al., 2015). Senescent microglial cells have been discovered to express aging-related phenotypes. Treatment of BV2 microglia with lipopolysaccharide resulted in growth arrest, SA-β-Gal up-regulation, and senescence-associated heterochromatin foci (SAHF) (Yu et al., 2012). In another study, microglia from aged mice exhibited increased production of the pro-inflammatory cytokines such as IL-6, IL-1β and TNF-α (Sierra et al., 2007), which were identified as the characteristics of senescent cells. Senescent microglia positive for p16Ink4a was found to accumulate in mice model of tau-dependent neurodegenerative disease, and their disruption greatly reduced the observed pathology (Krishnamurthy et al., 2004; Bussian et al., 2018). In AD patients, the human Alzheimer’s microglia (HAM) profile analysis reflected an enhanced human aging phenotype and unique transcriptional activation (Srinivasan et al., 2020).

Astrocytes are one of the most abundant cell types in the brain. They are responsible for homeostatic maintenance at all levels of the organization, from the molecular to the whole organ in the CNS (Verkhratsky & Nedergaard, 2018). Astrocytes exposed to various stresses lead to aging, which is characterized by the SA-β-Gal activity, growth arrest, increased expression of p16, p21, and p53, and SASP production (Bitto et al., 2010). With the increase in age, the number of p16^Ink4a^ positive astrocytes increases in human brain tissue (Bhat et al., 2012). Senescent astrocytes expressing SASP were enriched in the frontal cortex of patients with AD as compared to age-matched non-AD adults (Bitto et al., 2010). Genes related to neuronal development and differentiation were down-regulated in senescent astrocytes, while pro-inflammatory cytokines genes were upregulated (Boisvert et al., 2018). Meanwhile, senescent astrocytes increased the vulnerability of neurons to glutamate toxicity (Limbad et al., 2020), which is also one of the hypotheses of AD pathogenesis and the reason for cellular senescence.

In the CNS, myelin is an extension of oligodendrocytes concentrically wrapped around nerve axons (Kuhn et al., 2019). Functionally, myelin facilitates rapid transmission of axonal potentials and provides metabolic support to wrapped nerve axons. They are highly vulnerable to oxidative stress (Giacci et al., 2018). White matter changes in AD are thought to reflect both demyelination and axonal damage (Prins & Scheltens, 2015), while SA-β-Gal upregulation of oligodendrocyte in human cerebral tissue of white matter lesion (WML) may be evidence of oligodendrocyte senescence in AD (Al-Mashhadi et al., 2015). In the brains of human patients with AD and APP/PS1 mice model, OPCs with high expression of senescent phenotypes (p21, p16, and SA-β-Gal) were correlated with amyloid plaques, whereas in age-matched subjects without dementia, these senescent phenotypes were not obvious (Zhang et al., 2019). OPCs are very important for myelin regeneration following injury (Kuhn et al., 2019). Young mice had highly active myelination, while in aged mice, it was greatly inhibited, which coincides with spatial memory deficits (Wang et al., 2020). The impaired function of senescent OPCs may play a crucial role in disease progression, and reduced self-healing capacity could be due to the aging process and pathological factors such as Aβ deposition or NFT (Cai & Xiao, 2016; Zhang et al., 2019).

### 2.2. Aging neurons and neural stem cells (NSCs)

We can deduce from the relationship between age and AD that the function of neurons declines with age. In AD, the rate of human hippocampal neurogenesis slows, and many hippocampal neurons are lost. It was previously thought that aging only occurred in mitotic cells and that neurons are post-mitotic cells that do not experience cell senescence (Ishikawa & Ishikawa, 2020). This concept has changed in the last decade. An experiment conducted in 2012 proved that compared with the neurons in young mice, the aging phenotypes (SA-β-gal, p38^MAPK^, and IL-6) of neurons in old mice were greatly increased (Jurk et al., 2012). When *in vitro* primary rat hippocampal neurons were treated with AraC—a DNA intercalator—for 28 days, SA-β-gal activity increased, and p16 was upregulated (Ishikawa & Ishikawa, 2020). Other experiments also provided evidence of neural senescence, such as rising topoisomerase IIβ, SA-β-gal, and reactive oxygen species (ROS) (Bhanu et al., 2010; Geng et al., 2010; Dong et al., 2011). More evidence of senescent neurons and senescent NSCs in AD is being discovered. Data from the dentate gyrus of APP/PS1 transgenic mice model of AD showed upregulated p16 and increased SA-β-gal positive neural stem/progenitor cells (NSPCs) (He et al., 2013). Moreover, the number of NSCs in the brain of aging mice was observed to decrease sharply (Shook et al., 2012), and the activated capacity of quiescent neural stem cells (qNSCs) decreased with increasing age (Xie et al., 2020).

### 2.3. Aging BBB

BBB regulates the material exchange between blood and cerebral parenchyma, thereby maintaining the homeostasis of CNS. BBB mainly comprises brain capillary endothelial cells (BCECs), astrocytes, and pericytes. Among these, capillary endothelial cells play a major role in maintaining the integrity of BBB (Daneman & Prat, 2015). The integrity breakdown and increased permeability of BBB were observed in normal elderly, especially those with AD. This is conducive to the entry of peripheral toxic or inflammatory factors into the CNS. Compared with normal control groups, dynamic contrast-enhanced magnetic resonance imaging could detect significantly higher levels of gadolinium-diethylenetriamine pentaacetic acid (Gd-DTPA) in cerebrospinal fluid (CSF) after injection in AD cases (Yamazaki & Kanekiyo, 2017). The increase in blood-derived molecules and CSF/blood albumin ratio in the CNS of AD patients also verified this observation. Another review indicated reduced capillary density in the brain of aging and AD, which decreases the supply of essential nutrients and clearance of Aβ (Yamazaki et al., 2016). Impaired capillary endothelial cells may be the primary cause of dysfunctional BBB in AD, which can partly be attributed to cellular senescence. Some *in vitro* and *in vivo* modeling studies have indicated that senescent microvascular endothelial cells express a significant rise in SA-β-gal activity and p16^INK4a^ and p21 levels and are accompanied by the destruction of BBB integrity (Brown & Thore, 2011). There are few studies on senescent cells in BBB of neurodegenerative diseases, and more studies are expected to clarify the role of senescent capillary endothelial cells and other composing cellular senescence in BBB dysfunction. In this review, we have attributed the dysfunctional BBB to cellular senescence as aging BBB.

**Figure 1.**
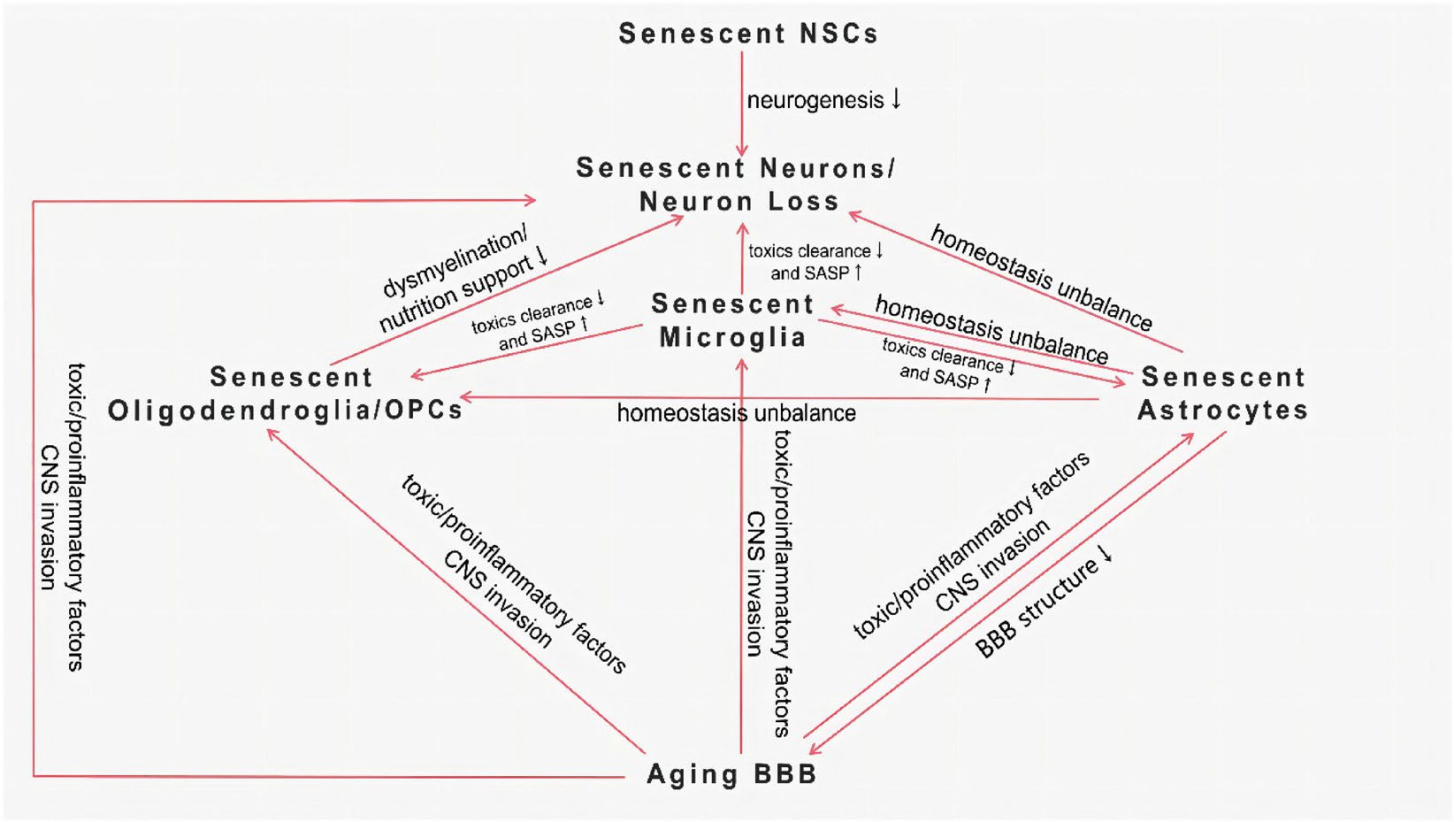
Interaction of senescent cells in the CNS.

Not much is known about the causal relationship between senescent cells and AD, but every type of senescent cell in the CNS and their interaction may be involved in AD pathogenesis. They constitute a complex interactive network (Figure 1) and finally lead to the degeneration and loss of normal neurons, leading to the onset and progression of AD. Future studies are expected to seek specific aging hallmarks and elucidate the interaction of different types of senescent cells in the cerebrum of AD.

## 3. Anti-aging as therapeutics for AD

At present, there are no therapeutics to effectively change the course of AD and prevent the disease progression. Although aducanumab-a kind of amyloid monoclonal antibody-has been approved by the Food and Drug Administration in June 2021 (Aducanumab, 2012), controversy persists because relevant analyses did not prove the efficacy and safety of aducanumab in improving the disease (Alexander et al., 2021; Knopman et al., 2021). Targeting Aβ agents conducted in preclinical, early, and late stages of AD did not show abundant clinical efficacy (Vaz & Silvestre, 2020). Besides targeting Aβ, tau-aimed treatments have received more attention in the last years; however, most anti-tau drugs were still in the early phase of clinical trials (Vaz & Silvestre, 2020). Many lines of evidence implicate that cellular senescence plays an important role in the pathogenesis of AD, so anti-aging treatment can be another option for DMTs.

### 3.1. Senolytics-based senescent cells clearance

The elimination of senescent cells has shown improvement in many age-related diseases such as renal dysfunction, idiopathic pulmonary fibrosis, nonalcoholic fatty liver disease, osteoarthritis, a decline in immune function with age, cardiovascular disease, sarcopenia, and so on (Baker et al., 2011; McHugh & Gil, 2018). The effort to find senolytics started in 2005 and more than a dozen kinds of senolytic drugs (Table 1) have been identified by targeting senescent cells’ anti-apoptotic pathway to induce apoptosis in senescent cells (Prata et al., 2018; Kirkland & Tchkonia, 2020).

**Table 1.** Senolytics and related anti-aging experiments.

Senolytics	Target	Model of experiment
Dasatinib	Ephrins dependent receptor	AD phase II pilot (NCT04063124) (Kirkland & Tchkonia, 2020)
Flavonoid (Quercetin, Fisetin, Luteolin)	Multi targets (MAPK pathway, PI3K/AKT pathway, Bcl-2 family proteins, and so on)	Only Quercetin in AD phase II. Pilot (NCT04063124) (Kirkland & Tchkonia, 2020; Kopustinskiene et al., 2020; Lee et al., 2020)
Curcumin	Multi targets (Bcl-2, Bcl-XL, p38^MAPK^, and so on)	More than 250 clinical trials, including AD, cardiovascular diseases, cancer, and so on (Kunnumakkara et al., 2017).
Curcumin analog (EF24)	Bcl-2, Bcl-XL, Mcl-1	Senescent human umbilical vein endothelial cells (HUVEC), senescent fibroblasts, senescent pre-adipocytes, and senescent human renal epithelial cells *in vitro* (Li et al., 2019).
Piperlongumine	Caspase-mediated apoptosis	Parkinson’s disease animal model (Wang et al., 2016).
Navitoclax (ABT263)	Bcl-2, Bcl-XL	Pathological fibrosis *in vivo* and *vitro* (Mohamad Anuar et al., 2020).
A1331852 A1155463	Bcl-XL	Senescent HUVEC *in vitro* (Zhu et al., 2017).
HSP90 inhibitors	PI3K/AKT pathway	*Ercc1* ^−/−^ murine embryonic fibroblasts *in vitro*, *Ercc1* ^−/Δ^ mice *in vivo* (Fuhrmann-Stroissnigg et al., 2017).
FOXO4-DRI	FOXO4/p53 axis	Human testes samples, senescent chondrocytes *in vitro* (Bourgeois & Madl, 2018).
Nutlin3a	p53	Senescent retinal pigment epithelium cells in an animal model (Chae et al., 2021).
Cardiac glycosides	Na^+^/K^+^ ATPase, Bcl2 family proteins	Cell models (osteoarthritis, pulmonary fibrosis, SKHep1 liver cancer cells, A549 lung cancer cells) and aging mice (Guerrero et al., 2019; Triana-Martínez et al., 2019).
Fibrates	Peroxisome proliferator-activated receptor alpha (PPARα)	Chondrocytes from cartilage degeneration and osteoarthritis *in vitro*, clinical retrospective study (Nogueira-Recalde et al., 2019).
Senescence-specific killing compound-1 (SSK1)	p38^MAPK^	Senescent mouse embryonic fibroblasts (MEFs), senescent mouse lung fibroblasts, senescent human embryonic fibroblasts (HEFs), senescent HUVEC, lung-injured mice, aged mice (Cai et al., 2020).

**Reference annotations**:

* doi:10.1002/alz.12328, *doi:10.1038/nm.4000,

** doi:10.1002/adma.201801362,

** doi:10.1111/joim.13141,

** doi:10.1002/advs.202004929,

* doi:10.1101/gad.343129.120,

* doi:10.1056/NEJMra0912273.

Nanoparticles-based anti-aging treatment is a promising therapeutics for AD.

The removal of senescent cells has been shown to improve pathology or symptoms in Alzheimer’s disease patients and experimental models. A tau-dependent neurodegenerative disease mouse model was used in the study, which accumulated p16INK4a positive senescent astrocytes and microglia (Bussian et al., 2018). In these mice, genetic methods (INK-ATTAC transgene and AP20187 administered) and senolytic ABT263 (navitoclax) were used to remove senescent astrocytes and microglia could prevent gliosis, hyperphosphorylation of soluble and insoluble tau, and degeneration of neurons in the cerebral cortex and hippocampus (Bussian et al., 2018). Similarly, other senolytics compounds-dasatinib and quercetin (D + Q)-applied for the intermittent treatment lasting up to three months to clear senescent cells associated with tau aggregation, significantly alleviated pathological characteristics, including the number of cortical neurons containing NFTs, ventricular pathological enlargement, and brain cortical atrophy (Musi et al., 2018). However, that study did not observe improvement in cognitive-related symptoms by clearing senescent cells in the mice model and is expected to be verified in later studies. Different from tau dependent model, D + Q removed senescent OPCs that highly expressed p16 induced by Aβ in short-term treated APP/PS1 mutant AD mice, reduced neuroinflammation and Aβ plaque size, and in the long-term intermittent treatment, it alleviated learning and memory deficits (Zhang et al., 2019). D + Q, as the most representative senolytics, could recruit phase II pilot (NCT04063124) volunteers to determine this drug effect in human patients by monitoring tau protein, amyloid, IL-6, and p16 level in the CSF and action and cognitive tests. To our knowledge, except for navitoclax and D + Q, other senolytics are not yet in use in AD models *in vivo* or *in vitro*. However, existing experiments have proved that senolytic drugs, which can selectively target senescent cells more than healthy cells, are effective pathogenesis-based DMTs modality for AD. Studies have focused on finding new therapeutic targets for senescent cells and developing pertinent therapeutic agents. Recently, urokinase plasminogen activator receptor (uPAR) was identified as a protein widely induced on the surface of senescent cells and showed that chimeric antigen receptor (CAR)T cells targeting uPAR could effectively and safely eliminate senescent cells around liver fibrosis (Amor et al., 2020). Limited studies of anti-aging therapeutics have demonstrated great potential for treating AD, but they are still far from clinical application.

### 3.2. Autophagy-base strategies

Autophagy is an important lysosomal degradation pathway mediated by several autophagy-related (ATG) genes, stabilizing cytoplasmic components, including degraded, misfolded, or aggregated proteins and damaged organelles promote the maintenance of cellular homeostasis (Glick et al., 2010). Autophagy capacity and ATG gene expression and protein levels decrease with age; genetic experiments of various model organisms have revealed that aging is directly related to different ATGs, and overexpression of a single ATG gene has been shown to prolong the life span of neurons (Hansen et al., 2018). The aging rodent brain showed notably increased mammalian target of rapamycin (mTOR)C1 activity and overall decreased ATG protein levels (Ott et al., 2016). In addition, down-regulation of ATG genes, including atg-5, atg-7, and becn-1 has also been observed in the aging human brain (Lipinski et al., 2010), indicating decreased autophagy. Similarly, mice with deficient ATG genes showed spontaneous and accelerated neurodegeneration and massive neuronal loss in the CNS cortex (Hara et al., 2006; Komatsu et al., 2006). Another report indicated that, except for neurons, autophagy of glial cells plays a critical role in the clearance of Aβ (Ries & Sastre, 2016), which can explain how impaired autophagy of glial cells affects the survival of nearby cerebral cells and induces the senescence and dysfunction of adjacent neurons. Thus, the decline in autophagy function, subsequent decrease in protein homeostasis, and increased protein toxicity over time are usually considered to contribute significantly to the development or progression of diseases, including neurodegenerative diseases. Autophagy may be reduced in AD and promote the accumulation of intracellular and extracellular Aβ (Pickford et al., 2008). The dysfunction of the lysosomal autophagy system was found to help the formation of tau oligomers and insoluble aggregates (Hamano et al., 2008). In contrast, stimulation of autophagy eased neurodegeneration owing to Aβ accumulation and Tau denaturation (Watanabe et al., 2020). Reducing mTOR levels and signaling by removing one copy of the mTOR gene led to increased induction of autophagy and improvement in AD-like changes, including cognition and pathological defects (Caccamo et al., 2014). Therefore, the restoration or promotion of autophagy function has been proposed as a promising method for delaying age-related diseases, including AD. Potential strategies included pharmacological treatments using mTORC1 inhibitors (rapamycin (Kaeberlein & Galvan, 2019), rapamycin analogs (Fanoudi et al., 2018), memantine (Son et al., 2016), latrepirdine (Steele & Gandy, 2013), and resveratrol (Kou & Chen, 2017)), activating AMPK (resveratrol (Kou & Chen, 2017), metformin (Piskovatska et al., 2020), RSVA314 (Vingtdeux et al., 2011), and RSVA405 (Sarkar et al., 2005)), inhibiting inositol monophosphatase (lithium and (Sarkar et al., 2005)), other pathways (Zhang et al., 2017) (GTM-1, carbamazepine), and non-drug methods (Escobar et al., 2019) such as caloric restriction, physical exercise, etc. Besides aiming signal pathways for autophagy, an acidification disorder was observed in lysosomes of senescent cells. This disorder resulted in neurodegeneration with cognitive impairment (Colacurcio & Nixon, 2016). The use of acidic nanoparticles targeted toward lysosomes could restore lysosomal acidification in the context of presenilin-1 mutation (Lee et al., 2015). Interestingly, the genome-wide analysis revealed that the autophagy level changed in different AD periods (Lipinski et al., 2010). From this analysis, autophagy transcription may be upregulated in the early stage of AD, which may be attributed to the protective changes in the body. This is not contradictory to the treatment of enhanced autophagy. However, the specific mechanism of changes in autophagy at different disease stages still needs to be further clarified, and therapeutic schedules targeting autophagy in different stages are required.

### 3.3. Anti-inflammation strategies

As mentioned above, senescent cells can actively secrete SASP to change their microenvironment and lead to damage or aging of nearby cells. Many pro-inflammatory cytokines in SASP, such as the IL-1 superfamily, IL-6, IL-8, macrophage inflammatory proteins (MIPs), and so on, are the primary mediators of the harmful effects of senescent cells leading to chronic and low-level inflammation (Lopes-Paciencia et al., 2019; Birch & Gil, 2020). Multiple components of SASP can also induce senescence of adjacent cells in a paracrine manner, resulting in the *in vivo* transmission of senescence (Acosta et al., 2013). In addition to SASP, some reports have proposed chronic inflammation mediated by pathological changes representative of AD and the gut microbiota-brain axis as the central mechanism (Forloni & Balducci, 2018; Kinney et al., 2018; Sochocka et al., 2019). Therefore, anti-inflammatory therapeutics may be beneficial in delaying cellular senescence. The agents regulating SASP (often called senomorphics) may be regarded as an alternative to senolytics (Birch & Gil, 2020). Senomorphics can produce a marked effect in three ways: blocking SASP signaling cascades, preventing the secretion of SASP, and inhibiting the activity of single components of the SASP (Childs et al., 2017).

SASP signaling cascades include complex pathways, including the cyclic GMP-AMP synthase-stimulator of interference genes (cGAS-STING) pathway, the Janus kinase, and signal transducer, activator of transcription pathway, GATA binding protein 4, p38^MAPK^, inflammasome, mTOR, and heat shock protein 90(HSP90). Notably, most SASP regulators seem to converge to NF-κB and the CCAAT/enhancer-binding protein β (C/EBPβ) (Sun et al., 2018; Lopes-Paciencia et al., 2019; Birch & Gil, 2020; Song et al., 2020). Several SASP signaling cascade pathways coincide with autophagy or apoptosis pathways, so related drugs such as rapamycin, metformin, curcumin, and inhibition of HSP90 may play an anti-aging role through different mechanisms (Moiseeva et al., 2013; Shehzad & Lee, 2013; Liu et al., 2016, 2017). Experiments targeting SASP signaling cascades have demonstrated that blocking signaling cascades can reduce the secretion of pro-inflammatory cytokines and effectively improve the function of aging tissues and organs. The role of cGAS in cellular senescence has received increased attention in recent years. For example, A151, a kind of cGAS inhibitor, reduces the production of pro-inflammatory cytokines, the inflammatory response of the injured brain, and protects neural function (Li et al., 2020).

As a set of proteins, the production of SASP undergoes a series of processes, including post-translational processing and secretion, before it takes effect, so proteases may be deemed as the key to regulating the activity of SASP (Childs et al., 2017). For instance, the IL-1 family matures after being cleaved by different proteases and achieves the maximum immune-stimulating activity. Proteases such as granzyme B, calpain, and others that can contribute to the maturation of pro-inflammatory cytokine may be good therapeutic targets (Childs et al., 2017). Besides achieving maximum immunity, blocking the process of SASP secretion from senescent cells is another method, and one therapeutic target is the ectodomain shedding mediated by metalloproteinase 17(ADAM17) (Effenberger et al., 2014; Afonina et al., 2015). At present, there are only a few studies on the maturation and secretion mechanism of SASP in senescent cells. More direct evidence of *in vivo* and *in vitro* models is required to prove that the above two promising methods can be applied for the clinical treatment of AD.

In addition, the third approach can be aimed at SASP components. Two studies showed that intracerebroventricular (ICV) anakinra—an IL-1R antagonist—and tocilizumab—an antibody against IL-6R-injection could significantly alleviate cognitive impairment and histopathological changes in AD models (Elcioğlu et al., 2016; Batista et al., 2021). Other SASP components such as TGF-β are also optional therapeutic targets. Thus, targeting SASP has a tremendous promising future.

The gut microbiota-brain axis successfully links neuroinflammation with peripheral mild chronic inflammation. The effort to restore intestinal flora balance has demonstrated anti-inflammation, anti-aging, and other positive effects on neurological function, such as improved cognitive function in the AD mouse model (Sochocka et al., 2019). Referring to anti-inflammatory drugs, NSAIDs are inevitably mentioned. The initial clue about the relationship between NSAIDs and AD came from rheumatoid arthritis patients who have long used NSAIDs, in which group the incidence of AD was unexpectedly low (McGeer et al., 1990). Subsequent comprehensive analysis of seventeen epidemiological studies confirmed that long-term NSAID intake is a preventive factor of AD in the normal elderly population (McGeer et al., 1996). Some studies have also confirmed the preventive effect of NSAIDs (Zandi et al., 2002; McGeer et al., 2016). Paradoxically, another large randomized study revealed that low-dose aspirin usage did not lead to any cognitive improvement after five years in participants over 50 years of age (Price et al., 2008). The contradictory results may indicate that NSAIDs can only play a preventive rather than therapeutic role and should be used at least a few years before the onset of the disease (Zandi et al., 2002). However, NSAIDs have not been recommended as a preventive drug for AD.

**Figure 2.**
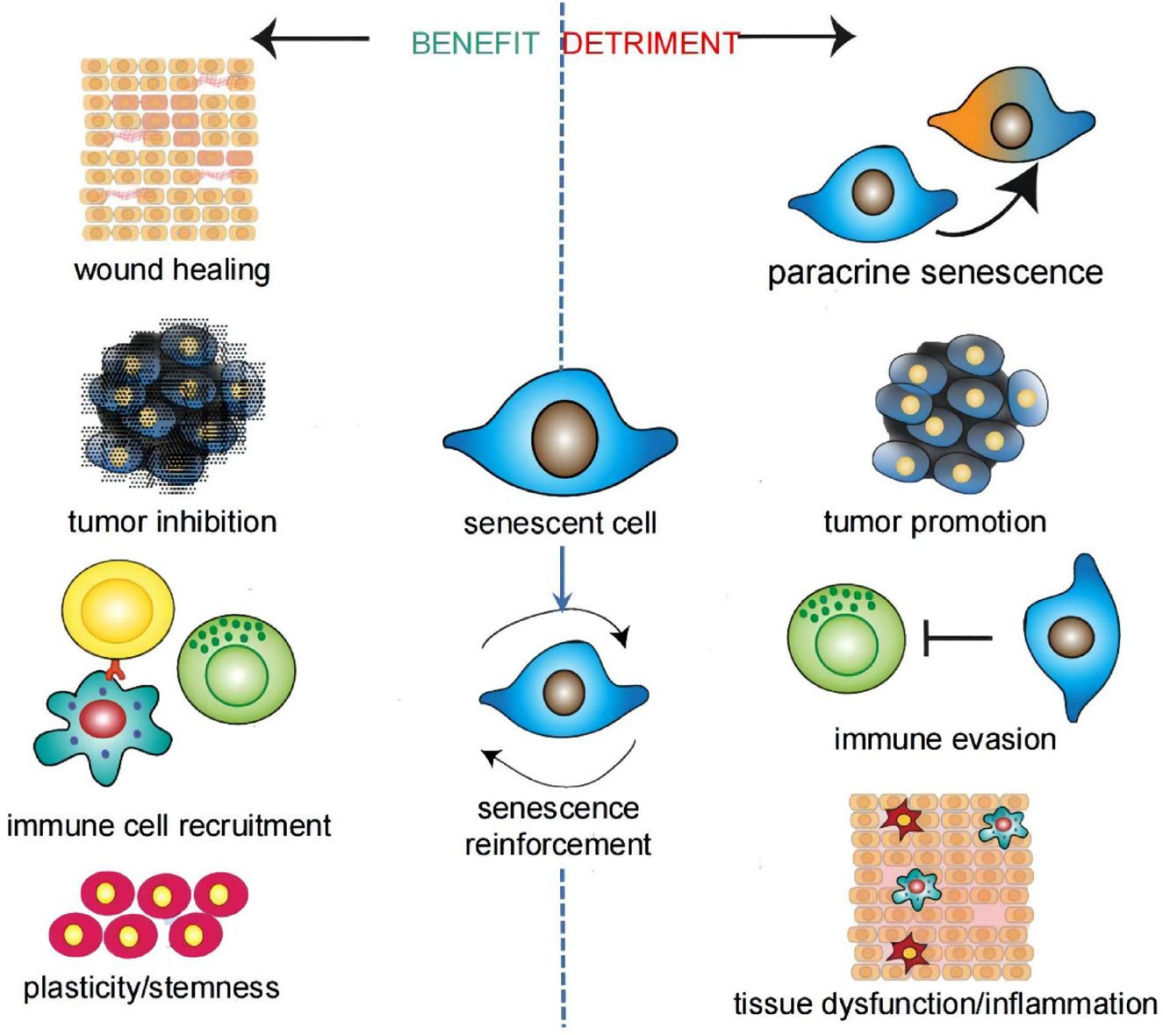
Benefit and detriment of senescent cells. Most of the effects are concerned with the SASP. Different inducements of aging, different types of senescent cells, and different aging stages give rise to these pleiotropic and contradictory effects. Adapted with permission (Birch & Gil, 2020). Copyright 2020, Cold Spring Harbor Laboratory Press.

Due to the diversity of cells in the CNS and the complexity of the aging process and SASP, more fundamental studies are essential. Senescent cells are a double-edged sword, and the benefit is that senescent cells have an important role in inhibiting tumors, tissue repair, and so on (Figure 2) (Song et al., 2020). Therefore, successful anti-aging treatment must avoid undermining related benefits.

### 3.4. Stem cell therapy

While the mechanism has not been fully comprehended, stem cell therapy has been shown to promote neurogenesis and improve cognitive function in AD rodent models (Duncan & Valenzuela, 2017; Han et al., 2020). Stem cell trials designed to treat AD can be divided into four types: embryonic stem cells (ESCs), mesenchymal stem cells (MSCs), induced pluripotent stem cells (iPSCs), and neural stem cells (NSCs).

MSCs, which can be obtained from umbilical cord blood or the Wharton jelly, have been most studied due to the accessibility and relative ease of manipulation. MSCs injection has been proven to reach the brain, differentiate into neuron cells, and boost the growth and differentiation of local stem and progenitor cells in the hippocampus by activating the Wnt pathway in rodent models (Han et al., 2020). Although the low rate of neuronal differentiation limits direct neurogenesis of MSCs, many other mechanisms have also been proposed that can be neuroprotective, including reducing Aβ deposition and plaque formation, inhibiting Aβ- and tau-related neuron death, upregulating anti-inflammatory cytokines, and reversing inflammatory reaction (Duncan & Valenzuela, 2017; Han et al., 2020). At present, MSCs are the only stem cell experimented with in AD therapy in humans and in a clinical trial (Kim et al., 2015). The feasibility and safety of MSCs injection in human brains in AD patients have been confirmed, although no significant clinical effects on cognitive decline have been observed.

NSCs reside in the dentate gyrus of the hippocampus and subventricular zone of the lateral wall of the ventricle in adults and can derive all types of nerve cells. Human neural stem cells (hNSCs) can migrate and differentiate into neurons and glial cells when transplanted into the lateral ventricle of the brain of AD mouse models (Duncan & Valenzuela, 2017). Similar to MSCs, although the low differentiation rate of neurons also limits the neurogenic effect of NSC, its secretion of nerve growth factor (NGF), brain-derived neurotrophic factor (BDNF), and anti-inflammatory cytokines may have great therapeutic potential to increase the density of neurons, synapses, and nerve fibers and reduce AD pathology to improve cognitive impairment in AD (Duncan & Valenzuela, 2017; Han et al., 2020). In aging mice transplanted with NSCs over-expressing choline acetyltransferase, improvement of cognitive function was observed, and the integrity recovery of cholinergic neurons in these mice was attributed to the increase in BDNF and NGF (Park et al., 2013).

ESCs is totipotent stem cell. HESCs transplantation can effectively mitigate the development of radiation-induced cognitive impairment in athymic nude rats (Acharya et al., 2009). However, direct transplantation of ESC into AD animal models leads to teratoma rather than neuron formation and is the primary limitation of the clinical translation. To circumvent this limitation, another tactic involves inducing ESC to differentiate into NSCs, MGE-like progenitor cells, or basic forebrain cholinergic neurons (BFCNs). In AD rodent model experiments, these cells can differentiate into cholinergic neurons and γ-aminobutyric acid neurons after transplantation to improve cognitive function (Duncan & Valenzuela, 2017; Han et al., 2020). Meanwhile, abnormal immune reaction, rejection, and ethical consideration are the other limiting factors.

The use of iPSCs, induced by upregulating designated transcription factors in mature somatic cells, is an emerging technology. iPSCs have similar morphology and differentiation ability as those of ESCs (Takahashi & Yamanaka, 2006). Therefore, their application modes and limiting factors in AD treatment may also be homologous. Nevertheless, compared with ESCs, iPSCs can come from autologous mature somatic cells, thus avoiding immune rejection and ethical disputes. However, iPSCs from patients with AD may express related neuropathological phenotypes such as high levels of Aβ, increased abnormal phosphorylated tau protein, and shortened nerve fibers after differentiation into neurons (Duncan & Valenzuela, 2017; Han et al., 2020). Thus, iPSCs are currently widely used to build models of AD rather than treatments. In recent years, the development of advanced gene transduction and editing technologies has expanded the application potential of iPSCs (Brookhouser et al., 2017). By which, iPSCs can differentiate into normal neurons used for transplantation.

## 4. Application of nanoparticles

The BBB is the brain’s most important physiological barrier. On the one hand, BBB protects the CNS from toxic substances in the environment. On the other hand, it prevents drugs from entering the brain effectively (almost all macromolecule drugs and 98% small molecule drugs). BCECs are the primary structures that lead to BBB function. Unlike peripheral capillary endothelial cells, which have high permeability, BCECs have tight junctions that limit the paracellular transport of compounds from the blood to the brain. Tight junctions also cause extremely high transendothelial resistance (TEER), which inhibits the passive diffusion of chemicals (Gao, 2016; Ruan et al., 2021). Pericytes and astrocytes are the other two types of BBB cells. They contribute to the integrity of the BBB by embedding within and coating the basement membrane, as well as inducing tight junction protein formation (Gao, 2016; Ruan et al., 2021). Only lipophilic drugs with a molecular weight of less than 500 Da can freely cross the BBB (Gao, 2016). As previously stated, the integrity of the BBB is compromised, and the permeability of the BBB is increased in the pathological changes of AD. However, vascular changes such as endothelial degeneration, disrupted transporter expression, perivascular accumulation of toxic products, and inflammation continue to obstruct drug delivery to the CNS (Sweeney et al., 2018). There are two approaches to delivering drugs into the CNS: bypassing the BBB or crossing it.

### 4.1. Strategies of overcoming the BBB

#### 4.1.1. Bypassing the BBB

The two main methods for bypassing the BBB are directly delivering drugs into the CNS and intranasal administration (Wong et al., 2019; Binda et al., 2020). Direct drug delivery into the CNS includes invasive procedures such as ICV administration, intrathecal administration, and others. ICV administration entails drilling a hole in the skull and injecting drugs directly into the intracerebroventricular space. Due to the rapid renewal of CSF and the blood-CSF barrier, however, less than 1–2% of the administered drug can slowly diffuse into the brain parenchyma from ICV in a single injection (Furtado et al., 2018). Therefore, agents flowing into the subarachnoid space with CSF from ICV and being absorbed via leptomeningeal transport may be the main mechanism consistent with the absorption pathway of intrathecal administration (Papisov et al., 2013; Atkinson, 2017). Intrathecal administration, which involves injecting drugs into the subarachnoid space of the spinal cord via lumbar puncture, is the most practical and least invasive method used in the clinic. Agents administered via lumbar puncture diffuse from lumbar CSF to intracranial CSF via pulsatile mixing, enter the periarterial spaces with active CSF pumping, and then move into the brain parenchyma (Papisov et al., 2013). In CFS, the duration of agents at therapeutic concentration levels influences the therapeutic effect. Therefore, using a continuous micro-pump to keep drugs in the CSF may improve the therapeutic effect. However, invasive procedures may have limitations such as an injury during the procedure, infection, and poor patient acceptance.

Intranasal administration is a noninvasive method of circumventing the BBB. Intranasal administration is the shortest and most efficient route for drugs to enter the CNS (via extracellular diffusion, intraneuronal transport by the olfactory sensory neuron or the trigeminal nerve). Other advantages include rapid absorption, rapid onset, avoidance of first pass metabolism, and high patient acceptance (Gao, 2016; Furtado et al., 2018). However, the high solubility of drugs (due to the limitation of a single dose), nasal epithelial barriers (only 5% of the total nasal surface area in humans belongs to the olfactory surface area), skilled operations (agents must be sprayed deep into the nasal cavity), and other factors pose challenges (Furtado et al., 2018). Many absorption enhancers or modulators, as well as nanoparticles, have been developed to address these challenges (Gao, 2016).

#### 4.1.2. Crossing through the BBB

**Figure 3.**
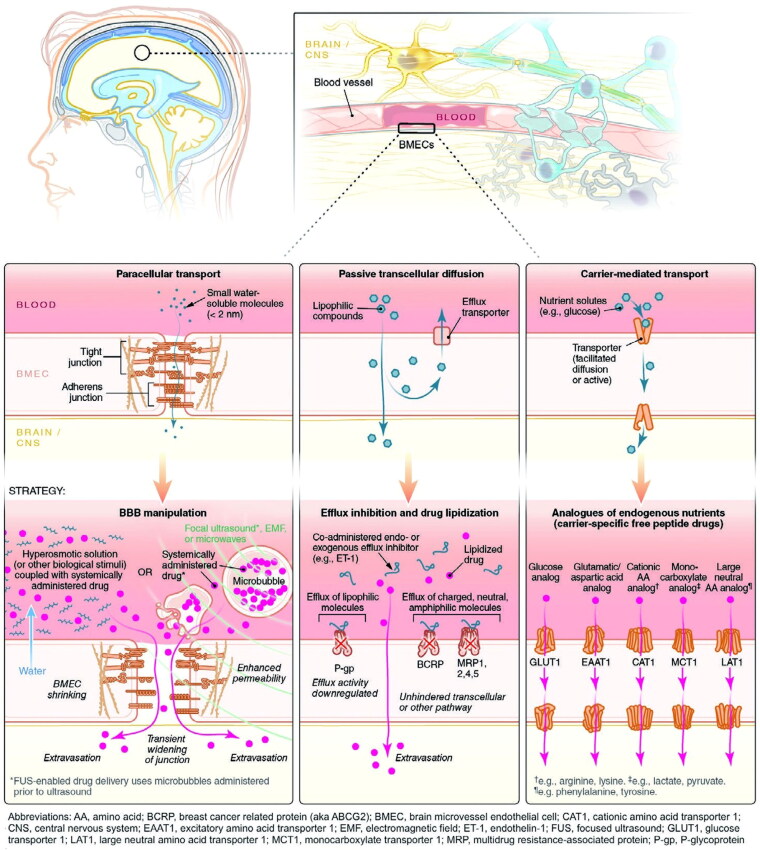
Six approaches by which agents cross through the BBB. Three such pathways include paracellular transport, passive transcellular diffusion, and CMT. Adapted with permission (Furtado et al., 2018). Copyright 2018, John Wiley and Sons.

**Figure 4.**
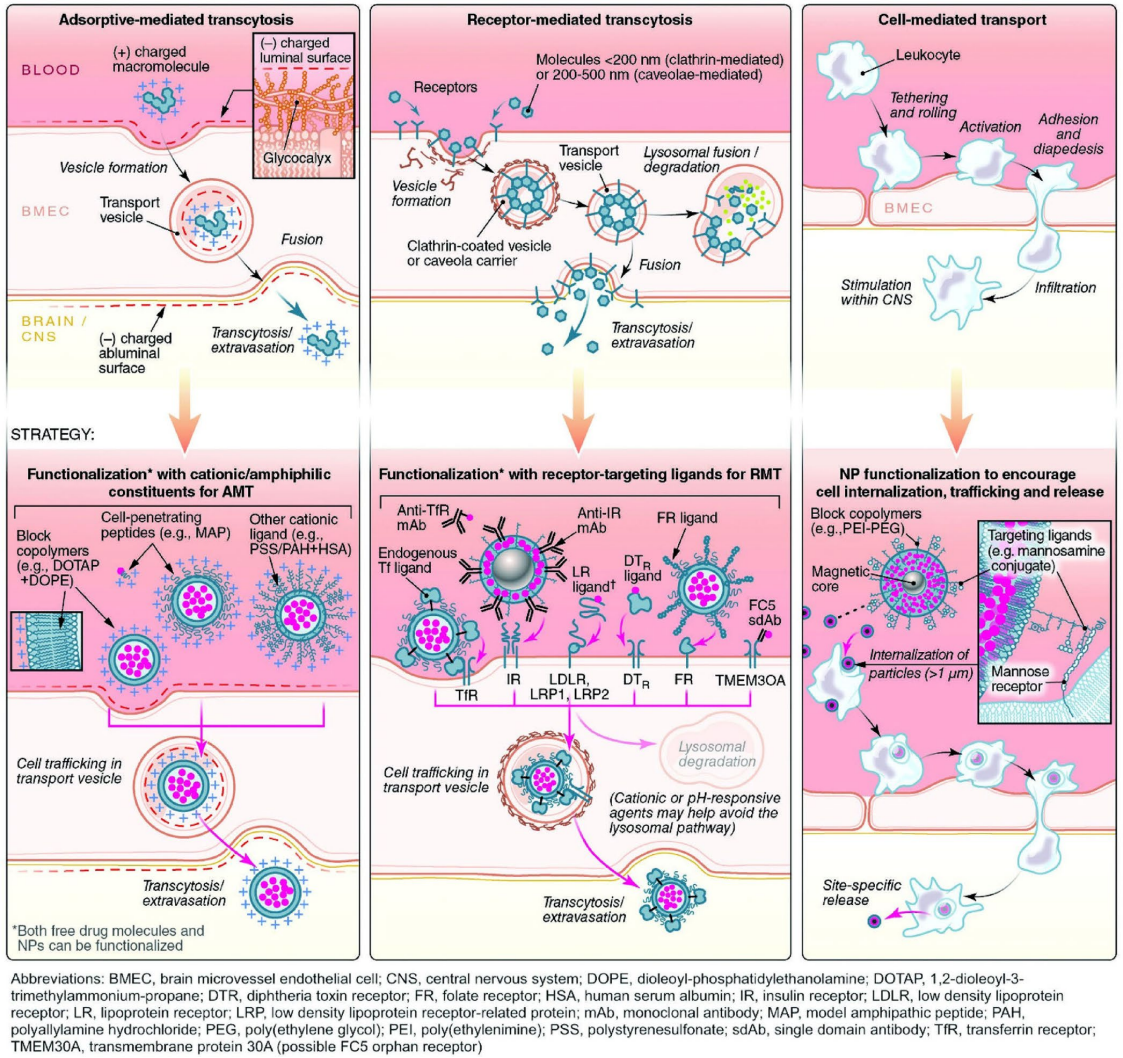
Six approaches by which agents cross through the BBB. Three other pathways include AMT, RMT, and cell-mediated transport. Adapted with permission (Furtado et al., 2018). Copyright 2018, John Wiley and Sons.

The most common route for drugs to enter the brain is via the BBB, which includes CMT, RMT, AMT, cell-mediated transport, paracellular transport, and passive transcellular diffusion (Figures 3 and 4). The majority of the nutrients required by the brain are transported from the blood to the CNS via a specific carrier or transporter. Glucose transporter-1 (GLUT1) and amino acid transporters are commonly high expressed on the BBB (Gao, 2016). CMT thus refers to assisting drug delivery through these specific influx transporters. RMT and AMT transport agents in a process known as transcytosis. Many receptors are highly expressed on the BBB, including transferrin (Tf) receptor, insulin receptor (IR), low-density lipoprotein receptor-related protein (LRP), diphtheria toxin receptor (DTR), and others (Gao, 2016; Ruan et al., 2021). The binding of corresponding ligands to these receptors causes RMT. RMT is currently the most successful and widely studied delivery method. Interestingly, in the context of AD, influx transport-related BBB changes include, but are not limited to, RAGE up-regulation and DTR up-expression, decreased IR sensitivity, and LRP and GLUT1 reduction (Sweeney et al., 2018). Tf receptor expression did not change significantly on the BBB in AD, and Tf receptor was also the most commonly targeted receptor for RMT (Gao, 2016; Sweeney et al., 2018). Other functional ligands have also been developed to successfully promote drug delivery. AMT is another electrostatic interaction-mediated transcytosis (creating a cationic surface charge on the cargo or conjugating the cargo with a positively charged moiety, such as a cell penetrating peptide) (Gao, 2016; Furtado et al., 2018). However, when compared to RMT, AMT has a lower affinity, and poor targeting of BBB and positively charged compounds may cause BBB damage (Furtado et al., 2018; Ruan et al., 2021). Cell-mediated transport is a distinct mode of transfer. Cell-mediated transport is distinct in that agents are designed to be absorbed by peripheral immune cells before entering the CNS via immune cell chemotaxis in the case of neuroinflammation (Batrakova et al., 2011). Cell-mediated transport has the potential to deliver drugs to the inflammatory microenvironment of senescent cells, but the compatibility of drugs and carriers is the primary issue to be addressed. Finally, tight junctions prevent nearly all drugs from entering the brain via paracellular transport. Currently, the BBB can be temporarily opened using chemical and physical methods such as borneol, activation of A1 or A2A adenosine receptors, and focused ultrasound (FUS), resulting in a significant improvement in drug delivery efficiency (Gao, 2016). However, short-term cognitive impairment and other adverse events following FUS in AD should be monitored.

### 4.2. Advantages of nanoparticles

Nanoparticles refer to nanomaterials that carry biomedical functional molecules by physical adsorption, encapsulation, molecular self-assembly, or chemical bonding. Nanoparticles are typically divided into several categories, including lipid-based NPs, polymeric NPs, inorganic NPs, nanogels, etc. Nanoparticles can be engineered to have varying sizes, shapes, hollow or solid structures, and tunable chemical, biological, magnetic, electronic, and optical properties to improve drug loading, stability, bioavailability, and targeting (Kim et al., 2010). Thus, nanoparticles can increase the efficiency with which agents enter the CNS and decrease consumption in peripheral circulation by targeting transportation. Nanoparticles, particularly those with specific functionalization, can play an important role in the first four pathways of BBB crossing.

### 4.3. Nanoparticles-based anti-aging agents

In this section, we mainly refer to some drugs that are currently considered to have anti-aging effects applied for AD. With the aid of nanoparticles, these anti-aging agents can improve the condition of AD more significantly via anti-inflammatory or alleviating pathological changes. Nanoparticles-based anti-aging treatments in AD are expected to exert promising anti-AD therapeutic efficacy in future studies.

#### 4.3.1. Lipid-based NPs

Lipid-based NPs are usually stable and nontoxic spherical vesicles composed of one or more phospholipid layers and are well suited to brain drug delivery applications. Liposomes and solid lipid NPs (SLNs) are the most common types. The core of SLNs is biocompatible lipids, which can solubilize lipophilic molecules, while liposomes have a hydrophilic core (Furtado et al., 2018). Lipid-based NPs have the features of low toxicity and immunogenicity. However, the stability of lipid-based NPs in the gastrointestinal tract and drug loading capacity should be further improved.

‘Naked’ liposomes can hardly carry drugs directly crossing through the BBB into the brain via passive transport. However, functionalized or modified liposomes to change the physical and chemical properties of the surface can bring the penetration capacity into full play by targeting transport; besides, these can also be designed to aim at specific targets related to AD (Ross et al., 2018). Curcumin-associated liposome delivery systems, for example, have a high affinity for Aβ plaques and, after being modified by PEGylated lipid coating, can attach anti-transferrin monoclonal antibodies for BBB transport mediation (Mourtas et al., 2014). In another study (Meng et al., 2015), a novel nanostructured lipid carrier (NLC), which was modified with lactoferrin (Lf) and loaded with curcumin (Cur-loaded Lf-mNLC), was designed for brain-targeted delivery. Lf-mNLC showed the maximum absorption of the brain capillary endothelial cells (which was 1.39 times higher than NLC) *in vitro* conditions, and Cur-loaded Lf-mNLC showed significant improvement in the pathological injury of neurons in rats. Another Lf functionalized liposome RMP-7-Lf-QU-LS, which is quercetin (QU) encapsulated liposome grafted with RMP-7 and Lf, could be recognized by capillary endothelial cells via Lf receptor (LfR) and enhanced the penetration capacity of QU with low cytotoxicity to BBB, as well as prevented Aβ caused neurodegeneration and improved the viability of neurons *in vitro* model (Kuo & Tsao, 2017). Researchers also designed quercetin- and rosmarinic acid-loaded liposomes with conjugated phosphatidic acid and apolipoprotein E (ApoE-QU-RA-PA-liposomes), which can effectively penetrate the BBB without damaging the BBB after ApoE modified and promote neurons recovery from Aβ induced neurotoxicity by degrading Aβ and anti-inflammation, both *in vivo* and *in vitro* (Kuo et al., 2020). The permeability of BBB of resveratrol-loaded SLNs functionalized by ApoE also increased significantly (1.8-fold higher) than non-functionalized SLNs. The entrapment efficiency of SLNs for resveratrol reached 90% and resveratrol was released in a controlled and linear manner at the target site (Neves et al., 2016). Other modes (insulin and insulin-like growth factors, low-density lipoprotein, cell-penetrating peptides, etc.) of functionalized lipid-based NPs had also been recommended (Ross et al., 2018).

In addition to encapsulating anti-aging agents into the carriers, conjugating curcumin on the surface of liposomes by covalent bonding showed high affinity toward amyloid deposits in APP/PS1 transgenic mouse models and postmortem brain tissue of AD patients. Further, it also strongly inhibited Aβ fibril formation (Lazar et al., 2013; Kuo et al., 2018). The analogous designs which can deliver an agent from BBB to a region of interest or target cells in the CNS—secondary targeting drug delivery—have also been extended to other types of nanoparticles.

#### 4.3.2. Polymeric NPs

Polymeric NPs are a series of promising delivery carriers with characteristics of high drug loading, controllable drug release, and ease of modification. There is a great variety of polymeric NPs, including nanocapsules, nanospheres, micelles, dendrimer, polymersomes, and polyplexes, and Polymeric NPs are composed of biodegradable or biocompatible polymers such as poly(lactic-co-glycolic acid) (PLGA), poly(alkyl cyanoacrylate), PEG, poly(amidoamine) (PAMAM) and so on. PLGA is the most commonly used to synthesize nanocapsules, while PAMAM is dendrimers. In one study (Tiwari et al., 2014), after 24 h of *in vitro* treatment with simple curcumin-loaded PLGA NP (cur-PLGA-NPs), most of cur-PLGA-NPs were internalized into neural stem cells and neurospheres. *In vivo*, cur-PLGA-NPs could cross the BBB of rats and then localized in the hippocampus after intraperitoneal administration. In the process, curcumin was sustained and slowly released from NPs. Besides, compared with the same doses of free curcumin, the curcumin level in the brain of the cur-PLGA-NPs treatment group increased by 2.1 to 2.8 times, and hippocampus and neuronal cell proliferation was enhanced too. Cur-PLGA-NPs also showed efficacy even at the dose of .5 mg after directional injection in the hippocampus, while a high dose of curcumin did not show significant enhancement of neurogenesis. Some existing and potential drugs beneficial to AD (galantamine, memantine, resveratrol, quercetin, dexibuprofen, etc.) encapsulated by polymeric NPs also showed a significantly elevated therapeutic index (Wong et al., 2019; Binda et al., 2020).

Similar to lipid-based NPs, polymeric NPs can also be functionalized to enhance penetration and targeting. Recent research showed conjugation of PLGA NPs with CRT peptide (a cyclic iron-mimic peptide that targets TfR) increased their permeation across the BBB (Huang et al., 2017). Curcumin-PLGA NPs conjugated with Tet-1 (a 12-amino acid peptide with the binding characteristics of tetanus toxin) can raise the uptake of the neuron (Mathew et al., 2012). G7(Gly-L-Phe-DThr-Gly-L-Phe-L-Leu-LSer(O-b-D-Glucose)-CONH2), B6 peptide (targeting TfR as a substitute for transferrin protein), and 83-14 monoclonal antibody (MAb) modified curcumin-PLGA NPs also showed high-performance in permeation of the BBB and potential application in the treatment of AD (Barbara et al., 2017; Fan et al., 2018; Kuo & Tsai, 2018). Researchers recently developed R@(ox-PLGA)-KcD, a novel nanocleaner. R@(ox-PLGA)-KcD significantly reduced the inflammatory microenvironment, Aβ and p-Tau burden in the brain, and rescued memory deficits in an AD mouse model. Special components and structure were credited with excellent treatment outcomes (Lei et al., 2021). R@(ox-PLGA)-KcD is made up of PLGA linked to ROS-responsive Polyol-ox (made from inositol and oxalyl chloride) and then loaded with rapamycin (promoting autophagy). KLVFFC and C-Acp-Cyclo (CDAGRKQKC) were linked with DSPE-PEG2000-Mal and DSPE-hyd-PEG5000-Mal, respectively, to form DSPE-PEG2000-KLVFF and DSPE-hyd-PEG5000-DAG. They were then modified on the core’s surface to form an integrated nanocleaner. R@(ox-PLGA)-KcD can specifically target BBB at lesions by targeting its receptor connective tissue growth factor C-Acp-Cyclo (CTGF). R@(ox-PLGA)-KcD cleaved DAG via lysosome in endothelial cells, and the remainder of the nanocleaner [R@(ox-PLGA)-K] was exocytosed into the brain parenchyma (Lei et al., 2021). Other polymers, in addition to PLGA, have recently received a lot of attention. ApoE3-mediated poly(butyl) cyanoacrylate nanoparticles with curcumin (ApoE3-C-PBCA) are an effective drug delivery system. The increased uptake of curcumin and the synergism between ApoE3 and curcumin were attributed to ApoE3-C-enhanced PBCA’s neuroprotection and anti-amyloidosis (Mulik et al., 2010). Furthermore, Ab peptide (KLVFFAED) modified micelles containing curcumin (APLB/CU) can cross the BBB and be actively internalized by neurons or activated microglia via RAGE. APLB/CU improved cognitive function by alleviating the microenvironment of APP/PS1 mice brain, such as protein plaque and neuroinflammation, via a transportation-mimicked pathway (Lu et al., 2019). Recently, a study linked polycaprolactone (PCL) terminus-modified PEG with RAP peptide (a specific ligand of RAGE) via ROS-sensitive sulfur ether linker (MAH-EDT) and then self-assembled with tacrolimus (FK506) and ibuprofen to form Ibu&FK@RNPs (He et al., 2022). After 21 days of Ibu&FK@RNPs administration, the synaptic density and water maze test results of AD mice were similar to the wild control group. Aβ burden and the activation of astrocytes in the Ibu&FK@RNPs group were 1.64 and 12.78 times lower than the untreated group, respectively. Changes in the Ibu&FK@RNPs group were all much better than those in Ibu&FK@NPs (non-RAP peptide modified) group (He et al., 2022). Thus, targeting RAGE has the potential to penetrate BBB efficiently.

Compared with the other nanoparticles, dendrimers have rich terminal functional groups that can provide multiple attachment sites for the conjunction of drugs and other targeted components, and chemical bonds can be hydrolyzed in cells, which is conducive to drug release (Al-Azzawi et al., 2018). Dendrimers are hyperbranched polymeric macromolecules with a dendritic structure. Non-modified PAMAM dendrimer had been proved to pass through impaired BBB induced by inflammation and then located in the Intracranial inflammatory microenvironment in rabbit models. Loading anti-inflammation drugs(N-acetyl-L-cysteine or tesaglitazar) can significantly enhance bioavailability and effectively inhibit inflammatory response (Kannan et al., 2012; DeRidder et al., 2021). In a recent study, a multi-target dendrimer, namely PBP, was composed of 8-arm hydroxylating PEG linked with ROS-sensitive alkynyl and then in conjunction with p-Nrf2. With the modification of the Ab peptide, they can effectively target the inflammatory microenvironment of AD to reduce inflammation and ROS, decrease the Aβ burden, and eventually improve cognitive functions in AD model mice (Liu et al., 2021). To overcome the main disadvantage of flurbiprofen—low brain permeability-applied for neuroinflammation, poly(Epsilon-Lysine) dendrimer was designed to increase the permeability of flurbiprofen to the BBB *in vitro* (Al-Azzawi et al., 2018). Dendrigraft poly-l-lysine (DGL) is used for gene delivery due to its high gene transfection efficiency (Cai et al., 2020). T7 peptide (HAIYPRH, targeting Tf receptor) was modified onto DGL with acid-cleavable long-chain PEG (D-DT) after Dp (an all D-amino acid inhibitor inhibiting tau phosphorylation) and Tet1 (a 12-amino-acid peptide targeting neuron) were modified .onto DGL with short-chain PEG (D-DT) (D-DTCT7). Finally, static electricity was used to load the BACE1 siRNA onto the D-DTCT7 to synthesize D-DTCT7/siRNA (Cai et al., 2020). D-DTCT7/siRNA was shown to target the BBB and neurons, inhibit the formation of Aβ and p-Tau-related fiber tangles in neurons, and significantly improve cognitive impairment in AD mice. The superiority of dendrimers is apparent. However, with the more branches and larger molecular weight of dendrimers, although the drug loading increased, it was accompanied by the increased toxicity of carriers and a decrease in clearance rate and drug release rate (Al-Azzawi et al., 2018). Balancing the pros and cons requires further research.

#### 4.3.3. Inorganic NPs

Inorganic NPs include gold NPs (AuNPs), magnetic NPs (MNPs), silver NPs, titania NPs, ceramic NPs, and many others. Different kinds and sizes of inorganic NPs have different physicochemical properties. Compared with the first two types of NPs, inorganic NPs have been relatively rarely examined to treat AD. Based on the unique physicochemical properties (ultra-small size, inert chemical property, excellent biocompatibility, ease of functionalization), AuNPs can be a potential drug delivery carrier (Gao et al., 2017). *In vitro*, an experiment using Aβ(1–40) as an example revealed that large AuNPs (average diameter: 36.0 ± 3.0 nm, 18.1 ± 3.0 nm) coated by L-glutathione (GSH) accelerated the growth of Aβ fibrils, whereas smaller AuNPs (average diameter of 6.0 ± 2.0 nm) largely suppressed this process, while gold nanoclusters (AuNCs; average diameter of 1.9 ± 0.7 nm) showed much stronger inhibition effect toward Aβ fibrillation. Interestingly, complete inhibition can be observed when AuNCs concentration reaches 10 ppm (1 ppm = 1 µg·mL^–1^) or higher, and a similar inhibition effect of AuNCs was also observed for Aβ(1–42) (Gao et al., 2017). However, the study pointed out that AuNPs with a size of under 40 nm could cross the BBB most efficiently, while a size smaller than 4–5 nm possibly induces cytotoxicity by penetrating the nucleus and binding DNA due to very high surface area over volume ratios. Therefore, the size of AuNPs between 10 nm to 30 nm seems to be the ideal choice (Soenen et al., 2011).

AuNPs with smaller diameters have a stronger inhibition of Aβ fibrillation, but how to avoid cytotoxicity caused by too small size AuNPs, the surface modification should be considered. A study indicated that chiral L- and D-GSH coated AuNPs with diameters of 3.3 nm, namely L3.3 and D3.3, can be transported from the blood circulation to the brain by intravenous injection and reduced Aβ deposition without obvious cytotoxicity at the experimental dose (25 mg/kg), and D3.3 showed higher brain distribution and significant improvement in spatial learning and memory (Hou et al., 2020). In another study, the researchers grafted polyoxometalates with Wells–Dawson structure (POMD) which showed the inhibition effect on Aβ aggregation onto the surface of AuNPs (average diameter of 21.7 nm) and then conjugated with the N-acetyl-Cys-LPFFD peptide (N-Ac-CLPFFD)-an effective β-sheet breaker peptide-to form AuNPs@POMD-pep, it showed efficient blood-brain barrier penetration and synergistic effects in clearing Aβ and inhibiting Aβ-induced cytotoxicity and oxidase activity (Gao et al., 2015). Curcumin functionalized AuNPs (Au-curcumin) better enhanced the inhibition of Aβ fibrillation and disintegration of amyloid fibrils in a dose-dependent manner, compared with similar concentrations of curcumin or AuNPs alone (Palmal et al., 2014).

Besides AuNPs, inorganic NPs, including carbon NPs, Fe3O4 magnetic nanoparticles (MNPs), cerium oxide nanoparticles (CeO2-NPs), and selenium NPs have also been shown to cross the BBB, inhibit the aggregation of Aβ, disaggregate Aβ fibrils, alleviate Aβ-mediated peroxidase activity and cytotoxicity, and protect neurons and synapses (Bilal et al., 2020). Inorganic NPs are developing rapidly and have a great variety. Thus, inorganic NPs can act as multifunctional DDSs combined with anti-aging agents for AD.

#### 4.3.4. Nanogels

Nanogels are a class of nano level three-dimensional polymers with temperature and optical controllability composed of polymers through chemical and/or physical crosslinking and can improve drug release (Li et al., 2012). Compared with other nanoparticles, nanogels have high drug loading, sustained release, distinctive biocompatibility, and colloidal stability. They can also be surface-functionalized and effectively pass across the BBB (Shah et al., 2020). Insulin conjugated to the carboxyl-functionalized poly(N-vinyl pyrrolidone) nanogel system (NG-In) can cross the BBB mediated by InR *in vitro* model (Picone et al., 2016). NG-In protected insulin from protease degradation and allowed its safe delivery to the target site with the aim of anti-inflammation and anti-Aβ in the CNS (Picone et al., 2016). It also provided a new drug delivery system for AD. Although studies about nanogels focused on AD are relatively few now. However, many nanogels such as cholesterol ε-polylysine (CEPL) nanogel, poly (vinyl alcohol) nanogel, PEG-b-PMAA nanogel, polyethylene glycol conjugated nanogel have been used to load drugs for the treatment of intracranial infection, tumor, ischemia, and other diseases and significantly improved therapeutic effects (Shah et al., 2020). Nanogels are expected to be widely studied in the anti-aging treatment of neurodegenerative diseases.

### 4.4. Challenges and restrictions

Available evidence indicated that nanoparticles-based anti-aging agents have more robust stability and superior bioavailability *in vivo*. Nanoparticles have broad prospects as DDSs for AD. Unfortunately, anti-aging treatment for AD based on nanoparticles is still in the laboratory stage. Currently, the challenges and restrictions of nanoparticles are derived from their biocompatibility and stability.

When nanoparticles are used in CNS diseases, the primary concern is toxicity, particularly neurotoxicity. Neurotoxicity can be avoided by reducing the retention of nanoparticles and metabolites in the brain. Therefore, the inevitable question is how nanoparticles are removed from the CNS. Extracellular degradation by metabolizing enzymes, internalization and subsequent degradation by neurons and glial cells, entrance into CSF bulk flow and reabsorption into the bloodstream or cervical lymphatics, and brain-to-blood efflux via abluminal transporters (ATP-binding cassette transporter, P-glycoprotein) (Gao et al., 2017). Other constraints, such as the tendency of nanoparticles to aggregate abnormally in storage, lysosomal trafficking, protein corona formation, and rapid clearance from circulation, can be partially overcome by specific modified surface properties. Stabilizing coatings and cryoprotectants can be used to prevent abnormal aggregation. Coating with PEG, poloxamine 908, polysorbate 80, and other modified agents can reduce protein corona formation and increase metabolic time. Cationic nanoparticles, nanoparticles conjugated with acid-responsive compounds, and special ligands (FC5 sdAb and ligand targeting DTR) are lysosomal-escapable (Kannan et al., 2012; Barbara et al., 2017; Gao et al., 2017; Furtado et al., 2018).

Besides this, it is only in the first stage that the nanoparticles penetrate the BBB for AD treatment. More attention should be paid to formulating secondary or multi-targeting drug delivery systems for CNS diseases (Kannan et al., 2012; Barbara et al., 2017). Summarily, the toxicity and biocompatibility of NPs, the mode of drug release based on nanoparticles, transport of NPs oriented toward the lesion site, and metabolism or clearance of nanoparticles from CNS still need long-term and extensive studies.

## 5. Conclusion and future perspective

The etiology of AD has not been delineated completely. At present, the clinical treatment of AD can only improve relevant symptoms, and the studies have always been attempting to find treatment methods to cease or reverse the course of the disease. However, research on treatments such as Aβ monoclonal antibodies targeting pathological changes is not plain sailing. The primary problems are that the experimental results do not meet expectations, the indications are limited to the early or middle stage of the disease, and there are obvious side effects such as cerebral microbleeds and others.

Cellular senescence is an alternative choice to find the etiology of AD from another perspective and seek targeting therapeutics. While being promising, there are also many challenges. At first, most of the current studies on the senescent cells in the CNS focus on glial cells. Due to the peculiarity and complexity of the CNS, future studies are expected to verify the aging evidence of various neural cells and elaborate on the interaction between various senescent cells and the causal relationship between them and AD. Second, there are only a few studies on the anti-aging treatment of AD, especially when based on nanoparticles. Currently, studies on anti-aging therapeutics such as senolytics, enhancing autophagy, anti-inflammation, and stem cell therapy applied to AD models have proved its great potential as DMT, including alleviation of both pathology and symptom in AD. Although they are still in the phase of exploration and preliminary development, they may be promising clinical treatments in the near future. More studies are expected to combine anti-aging therapeutics with nanoparticles to treat neurodegenerative diseases such as AD in the future. Besides this, anti-aging therapy has been proved to improve the function of other tissues and organs, such as bone and joint degenerative diseases, cardiovascular diseases, muscle atrophy, and others. These diseases are common in the elderly. Given the age of onset of AD, perhaps CNS anti-aging therapeutics should not be treated in isolation but rather as part of systemic anti-aging therapeutics.

Finally, regarding the new DDSs, the nanoparticles are conducive to agent delivery, including the penetration of anti-aging agents through the BBB and targeted delivery, and can significantly improve pharmacokinetics and bioavailability. This review only discusses several main types of nanoparticles. Nanoparticles represent an emerging field with great potential, and numerous new types of nanoparticles are being explored. The specific surface modification of nanoparticles can make them efficiently cross the BBB and be located at different targets after entering the CNS. In another way, controllable drug release mode and the synergy between drugs, drugs, and nano carriers can enhance the bioavailability (Su et al., 2015; Huang et al., 2016). Furthermore, altering the drug administration modalities, such as oral administration, subcutaneous injection, and transdermal patching may accompany better medication compliance and safety. Nonetheless, its transition to clinical practice is still a long-term and complex task. Furthermore, because many types of tumor cells and immune cells have the proclivity and ability to migrate into the CNS, DDSs based on exosomes and apoptotic bodies derived from cytomembranes (tumor cells, immune cells, and others) have demonstrated significant advantages and potential in crossing the BBB. As a result, they have received increased attention in recent years, and they offer promising prospects for improving and perfecting AD therapeutics (Wang et al., 2021).

- **Executive Summary:**

Anti-aging treatment AND AD

- AD is heavy health and economic burden for the whole world.
- There are still no significantly effective DMTs for AD until now.
- Cellular senescence of the CNS is correlated with AD closely.
- Anti-aging can be a promising treatment for delaying and reversing the course of Alzheimer’s disease

Application of nanoparticles

- The BBB hinders the entry of many agents into the CNS.
- Nanoparticles can promote the stability and efficiency of anti-senescent drugs and lower their loss while penetrating BBB.
- Nanoparticles can also improve drug targeting.
- Lipid-based NPs, Polymeric NPs, Inorganic NPs, and Nanogels have been used widely for drug delivery of AD and are hopeful to enhance anti-aging treatment.
